# Clinical course of patients with hepatocellular carcinoma who experienced radiologic complete response after radioembolization

**DOI:** 10.3389/fonc.2024.1349632

**Published:** 2024-01-30

**Authors:** Sungmo Moon, Gyoung Min Kim, Jong Yun Won, Joon Ho Kwon, Juil Park, Kichang Han, Man-Deuk Kim, Hyung Cheol Kim, Dong Kyu Kim, Jin Young Choi

**Affiliations:** Department of Radiology, Research Institute of Radiological Science, Severance Hospital, Yonsei University College of Medicine, Seoul, Republic of Korea

**Keywords:** hepatocellular carcinoma, radioembolization, complete response, recurrence, predisposing factor

## Abstract

**Purpose:**

The purpose of this study is to elucidate the patterns of recurrence of hepatocellular carcinoma and to analyze factors that can predict recurrence after complete response to radioembolization.

**Materials and methods:**

A total of 289 consecutive patients who underwent radioembolization for the treatment of hepatocellular carcinoma at a single tertiary center were retrospectively reviewed. Baseline characteristics were collected and compared between the group showing complete response and the group showing noncomplete response. Data on recurrence status, time to recurrence, and the patterns of recurrence among the patients who showed radiologic complete response were collected. The group that maintained complete response and the group that experienced recurrence were compared, and the risk factors affecting recurrence were evaluated by logistic regression analysis.

**Results:**

The complete response rate was 24.9% (73/289). Age, sex, tumor markers, maximum tumor diameter, multiplicity, presence of vascular invasion, and target radiation dose were significantly different between the complete response and noncomplete response groups. The recurrence rate after complete response was 38.4% (28/73), and 67.9% (19/28) of recurrences occurred by 8 months after complete response. Eight patients who underwent resection/transplantation after complete response experienced no recurrence. Multiple tumors and a lower target radiation dose were independent risk factors of recurrence after complete response in the multivariate logistic regression.

**Conclusion:**

Hepatocellular carcinoma recurrence following complete response after radioembolization is not uncommon and frequently occurs within 1 year after complete response. Multiple tumors and a lower target radiation dose may be risk factors for recurrence.

## Introduction

Radioembolization using ^90^Y-microspheres has been employed for the treatment of malignant liver tumors, including hepatocellular carcinoma (HCC) and colorectal liver metastasis (1). Though radioembolization was introduced as a standard treatment option in recent guidelines (2), it is currently only recognized as a treatment for single HCC tumors with diameters of 8 cm or less and is widely used as a palliative treatment for intermediate or advanced stages or an alternative treatment when other standard treatment options are not feasible or have failed.

Studies on the factors that can predict the responses and prognoses of patients who have undergone radioembolization for the treatment of HCC have been actively conducted (3–9). Commonly mentioned prognostic factors include tumor size and number, portal vein invasion, serum albumin/bilirubin level, and radiation dose. Patients who experience good radiologic responses are expected to have better overall survival (OS) (10–12).

The role of radioembolization for bridging or down-staging before curative resection or transplantation has also been well described in published studies. Resection and transplantation are curative and the best options for the treatment of HCC, but suitable candidates are limited due to the risks of marginal hepatic function or the fact that the tumors are often at advanced tumor stages at the time of diagnosis. Thus, pre-planned and staged treatments featuring radioembolization followed by resection or transplantation can increase the possibility of cure and improve OS (13–16).

The best follow-up or additional treatment strategies for patients with HCC who show good responses after radioembolization for palliative care or as an alternative treatment when curative treatments are not feasible have not yet been well studied. HCC recurrence is very common, even in patients who have undergone curative resection or experience complete response (CR) after other locoregional treatments (17). Hence, predicting the possibility of recurrence after good responses and performing additional treatments at the appropriate times are important strategies for patients’ prognoses.

Therefore, the purpose of this study is to elucidate the patterns of recurrence of HCC and to analyze the factors that can predict recurrence after CR to radioembolization.

## Materials and methods

### Patients

This retrospective study was approved by the institutional review board, and the requirement for informed consent was waived. The database of consecutive patients who underwent radioembolization in a single tertiary center from May 2009 to August 2022 was reviewed. Patients with (1) cholangiocarcinoma or metastatic liver cancer, (2) a history of receiving other treatments for HCC, (3) extrahepatic metastasis, (4) planned combination treatment with radioembolization and systemic therapy, and (4) no imaging follow-up were excluded. HCC was diagnosed according to the European Association for the Study of the Liver Clinical Practice Guidelines: Management of Hepatocellular Carcinoma (18) with imaging findings or pathologic examination. Data on the patients’ age, sex, Child-Pugh classification, and tumor characteristics, including size, number, and presence of vascular invasion, were collected in the retrospective review of the electronic medical records and imaging findings.

### Treatment

Radioembolization was performed by two interventional radiologists with 12 and 24 years of experience in interventional oncology. A general work-up, including clinical evaluation, contrast-enhanced dynamic liver computed tomography (CT) or magnetic resonance imaging, and laboratory assessments, was conducted before treatment. Planning angiography and cone beam CT were performed to determine the tumor-feeding arteries through which to deliver the microspheres. Planar scintigraphy and single-photon emission CT were performed for adequate dose calculation. Both resin and glass microspheres were used mainly based on their availability during the treatment periods. Dose calculations were based on the partition model for resin microspheres and the Medical Internal Radiation Dose for glass microspheres, as recommended by the manufacturers. The microspheres were administered as selectively as possible to preserve unaffected liver tissue.

### Outcomes and follow-up data

Primary endpoint of this study was the factors affecting recurrence after CR and secondary endpoints include time to recurrence and pattern of recurrence. Imaging follow-up was performed at 1, 3, and 6 months after radioembolization followed by subsequent evaluations every 3–6 months according to the referring physician’s decision. Imaging responses and determinations of recurrence were made by consensus of the two radiologists according to the modified Response Evaluation Criteria in Solid Tumors (mRECIST) 1.1 (19). If there was disagreement, the senior radiologist made the final decision on the response after discussion. Data on recurrence status, time to recurrence, and the patterns of recurrence (i.e., local recurrence, intrahepatic distant recurrence (IDR), and extrahepatic metastasis) of the patients who showed radiologic CR were collected. For the patients who experienced recurrence, the first treatment for the recurrence was also searched.

### Statistical analysis

Student’s t-test was used for comparisons of continuous data, and the chi-square test or Fisher’s exact test was used for comparisons of categorical data. Kaplan–Meier curves were drawn to determine the time to recurrence after radiologic CR and to compare overall survival between the group of patients with sustained CR and the group of patients who experienced recurrence. To evaluate the factors affecting recurrence after CR, univariate and multivariate logistic regression analyses were performed to estimate odds ratios with 95% confidence intervals between the group of patients with sustained CR for over 1 year and the group of patients who experienced recurrence. P-values less than 0.05 were considered statistically significant for all analyses. SPSS ver. 26.0 was used for the statistical analysis.

## Results

**Figure 1:**
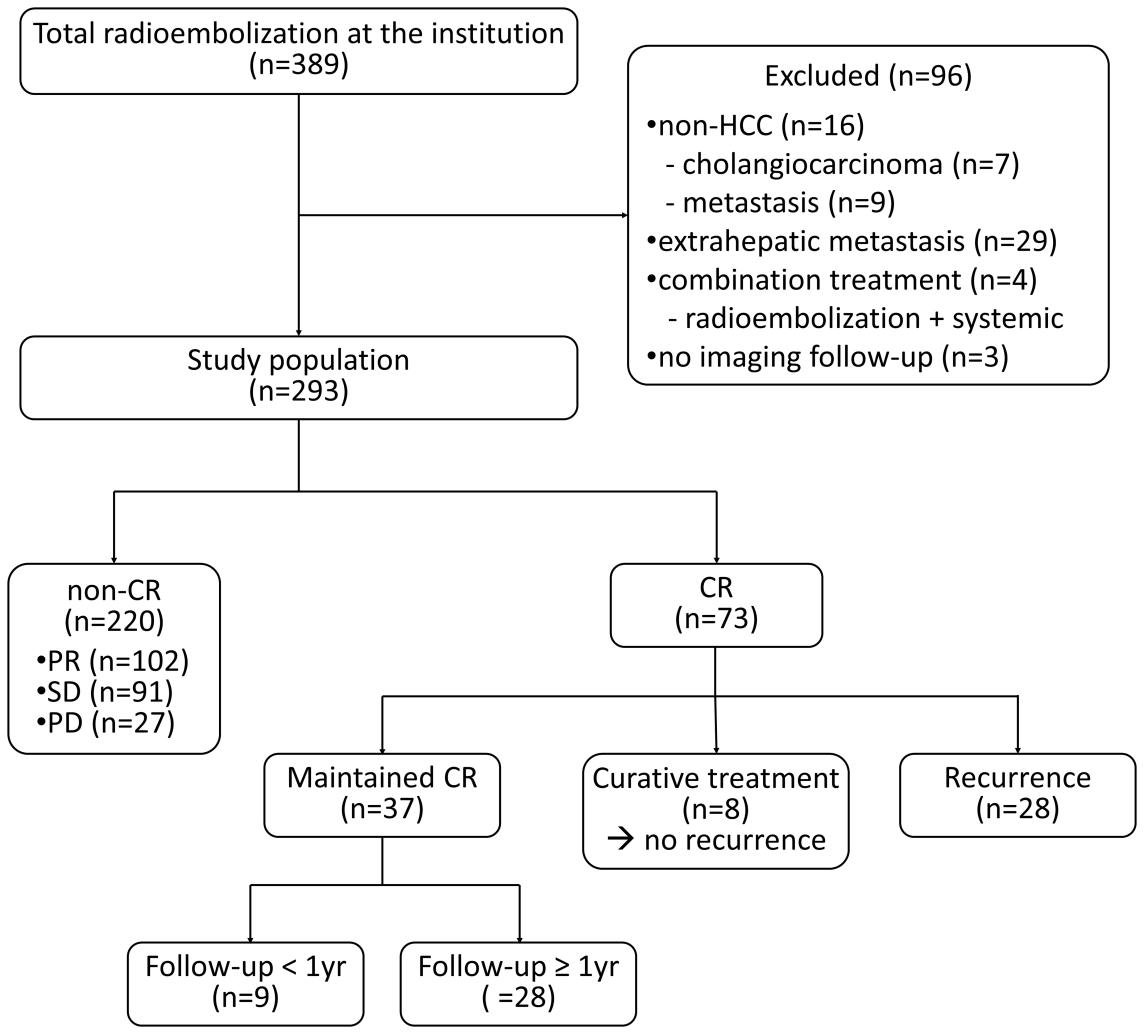
Study population.

During the study period, 389 patients underwent radioembolization at our institute. A total of 96 patients were excluded according to the exclusion criteria, and 293 patients were ultimately included in this study (**Figure 1**). The baseline characteristics are summarized in **Table 1**. The best response was CR, which occurred in 73 patients (24.9%); partial response (PR) occurred in 102 patients (34.8%), stable disease in 91 patients (31.1%), and progressive disease in 27 patients (9.2%). In the patient group demonstrating CR, the significantly prevalent factors included older age (mean 70.3 vs. 62.5 years), female sex (28.8% vs. 15.0%), lower alpha-fetoprotein level (median 15.8 vs. 108.9 ng/mL), smaller tumor size (mean 56.4 vs. 90.9 mm), single lesion (71.2% vs. 36.8%), absence of vascular invasion (89.0% vs. 72.3%), and higher radiation dose (mean 622.8 vs. 318.3 Gy). The median time to CR was 82 (19–352) days.

**Table 1:** Baseline characteristics of the study population.

		Total (n=293)	CR (n=73)	non-CR (n=220)	P-value
Age		64.4 (27–92)	70.3 (45–92)	62.5 (27–91)	<0.001
Sex	Male	239 (81.6%)	52 (71.2%)	187 (85.0%)	0.010
	Female	54 (18.4%)	21 (28.8%)	33 (15.0%)	
Child-Pugh class	A	274 (93.5%)	71 (97.3%)	203 (92.3%)	0.174
	B	19 (6.5%)	2 (2.7%)	17 (7.7%)	
AFP		48.0 (0.98–208190)	15.8 (1.3–120423.9)	108.9 (0.98–208190)	0.006
Maximum diameter (mm)		82.3 (14–255)	56.4 (18–131)	90.9 (14–255)	<0.001
Multiplicity	Single	133 (45.4%)	52 (71.2%)	81 (36.8%)	<0.001
	Multiple	160 (54.6%)	21 (28.8%)	139 (63.2%)	
Vascular invasion	Absent	227 (77.5%)	65 (89.0%)	159 (72.3%)	0.004
	Present	66 (22.5%)	8 (11.0%)	61 (27.7%)	
Microspheres	Resin	191 (65.2%)	46 (63.0%)	145 (65.9%)	0.672
	Glass	102 (34.8%)	27 (37.0%)	75 (34.1%)	
Mean dose		393.8 (51–2648)	622.8 (126–2648)	318.3 (51–2459)	<0.001

CR, complete response; AFP, alpha-fetoprotein.

Age, maximum diameter, and mean dose are presented with mean values (ranges).

Categorical variables are presented as numbers (percentages).

**Figure 2:**
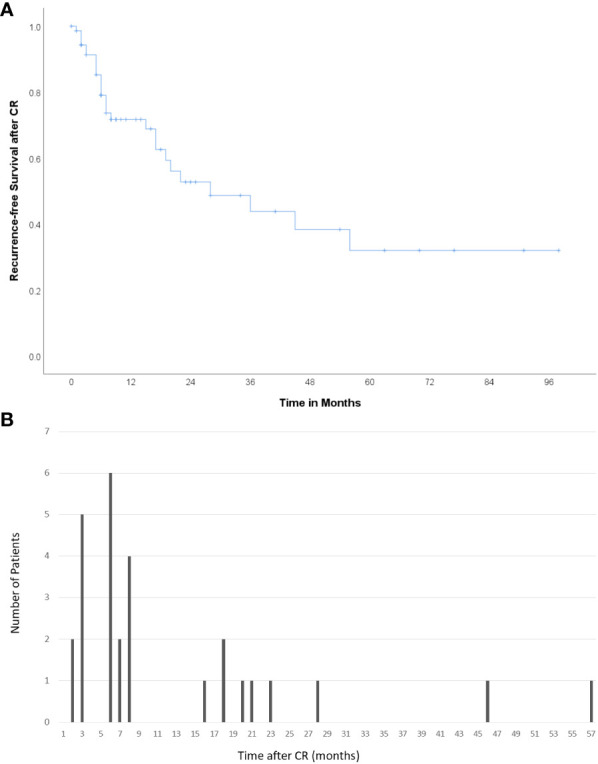
Recurrence after CR **(A)** Kaplan–Meier curve showing recurrence-free survival after CR. The median recurrence-free survival was 23.2 months. **(B)** Number of patients who experienced recurrence according to the time after CR. A total of 67.9% (19/28) of cases of recurrence occurred within 8 months after CR.

Among the 73 patients showing CR, eight (11.0%) underwent curative treatments, including resection (n=5) or transplantation (n=3). During the median follow-up period of 459 (89-2640) days after resection/transplantation, there was no recurrence among the patients who had undergone curative treatment. A total of 28 patients (38.4%) experienced recurrence and CR was maintained in the remaining 37 patients. The median follow-up period was 541 (87–4150) days, and the median recurrence-free survival after CR was 23.2 months (**Figure 2A**). The patterns of recurrence among the 28 patients included local recurrence around the treated lesion in 12 (42.9%), IDR in 10 (35.7%), extrahepatic metastasis in 4 (14.3%), and both IDR and extrahepatic metastasis in 2 (7.1%) patients. A total of 67.9% (19/28) of recurrences occurred within 8 months after CR (**Figure 2B**).

**Figure 3:**
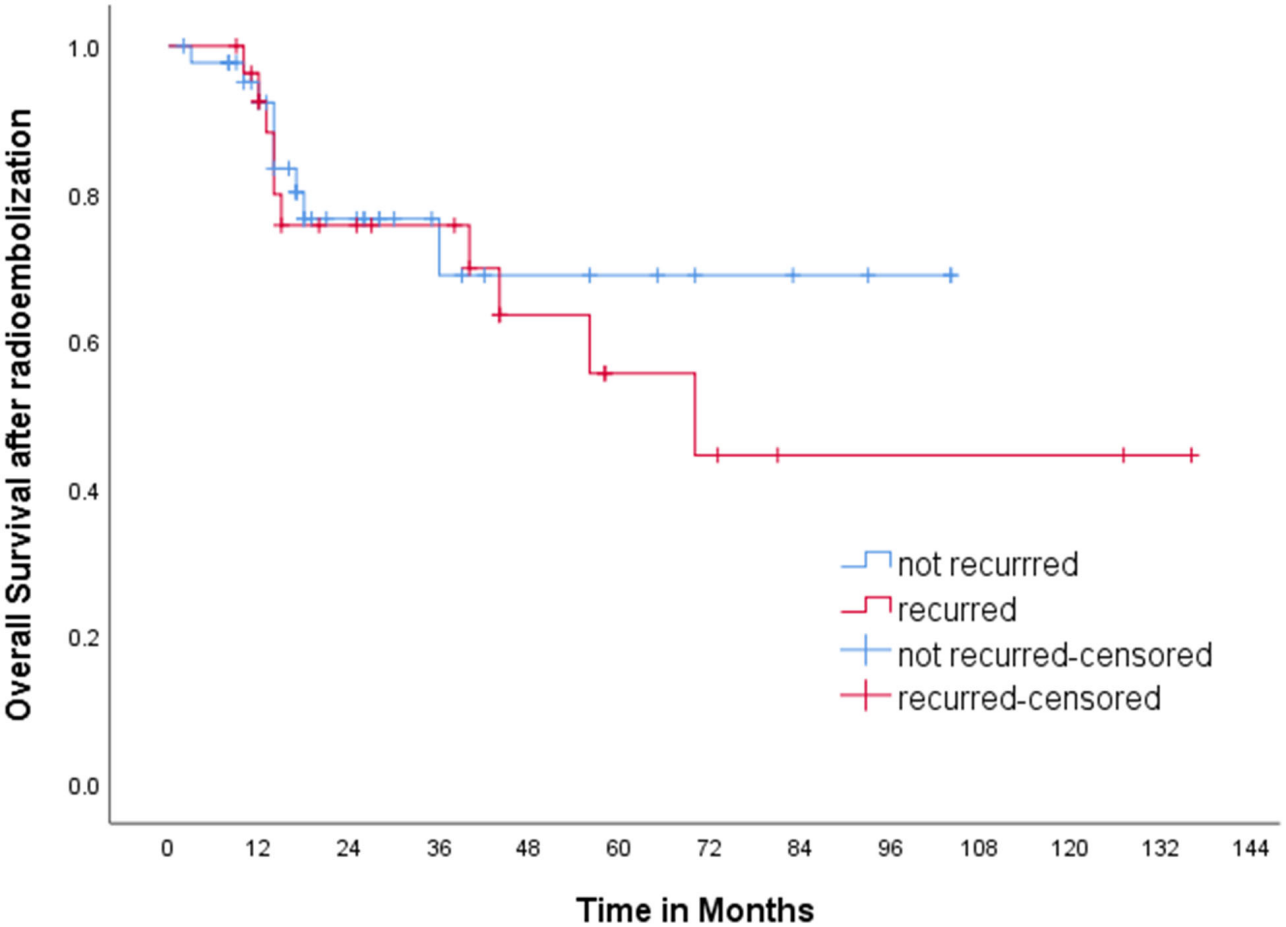
Kaplan-Meier curve for overall survival. Median overall survival of the group of patients who experienced recurrence was 72.0 months, while that of the group of patients with sustained CR was not reached (p=0.534).

Various kinds of treatment were applied for the recurrence, including chemoembolization (n=13), local ablation therapy (n=4), radiation therapy (n=3), resection (n=1), second radioembolization (n=1), and lenvatinib (n=1). Only supportive care was applied in three patients due to poor hepatic function or old age, and two patients were lost to follow after recurrence. Median overall survival of the group of patients who experienced recurrence was 72.0 months, while that of the group of patients with sustained CR was not reached (p=0.534) (**Figure 3**).

Excluding patients who received curative treatment and patients (n=8) with a duration of CR of less than 1 year (n=9), the characteristics of the patient group in which CR was maintained for more than 1 year (n=28) and the patient group in which recurrence was experienced (n=28) are summarized in **Table 2**. In the group in which CR was maintained, the age was significantly older and the mean radiation dose was significantly higher than in the group in which recurrence was experienced. Uni- and multivariate logistic regression analyses revealed that multiple tumors (odds ratio=5.976) and lower radiation doses of less than 400 Gy (odds ratio=4.713) were the only significant risk factors of recurrence after CR (**Table 3**).

**Table 2:** Difference between the group in which CR was maintained and the group in which recurrence was experienced.

		Total (n=56)	Maintaining CR (n=28)	Recurrence (n=28)	P-value
Age		70.6 (45–92)	73.6 (45–92)	67.6 (50–84)	0.035
Sex	Male	39 (69.6%)	18 (64.3%)	21 (75.0%)	0.562
	Female	17 (30.4%)	10 (35.7%)	7 (25.0%)	
Child-Pugh class	A	54 (96.4%)	26 (92.9%)	28 (100.0%)	0.491
	B	2 (3.6%)	2 (7.1%)	0 (0.0%)	
AFP		24.1 (1.3–120423.9)	7.0 (1.3–120423.9)	107.6 (1.3–27993.1)	0.262
Maximum diameter (mm)		59.1 (18–131)	55.1 (18–110)	63.0 (37–131)	0.148
Multiplicity	Single	41 (73.2%)	24 (85.7%)	17 (60.7%)	0.068
	Multiple	15 (26.8%)	4 (14.3%)	11 (39.3%)	
Vascular invasion	Absent	49 (87.5%)	25 (89.3%)	24 (85.7%)	1.000
	Present	7 (12.5%)	3 (10.7%)	4 (14.3%)	
Microspheres	Resin	37 (66.1%)	18 (64.3%)	19 (67.9%)	1.000
	Glass	19 (33.9%)	10 (35.7%)	9 (32.1%)	
Mean dose		609.2 (126–2648)	765.6 (140–2648)	452.7 (126–2112)	0.029

CR, complete response; AFP, alpha-fetoprotein.

Age, maximum diameter, and mean dose are presented with mean values (ranges).

Categorical variables are presented as numbers (percentages).

**Table 3:** Uni- and multivariate logistic regression analyses for the evaluation of risk factors of recurrence after CR.

		Univariate analysis		Multivariate analysis	
		Odds ratio (95% CI)	P-value	Odds ratio (95% CI)	P-value
Sex	Female				
	Male	1.398 (0.329–5.945)	0.650		
Age	<65 years				
	≥65 years	0.203 (0.564–14.852)	0.203	3.244 (0.779–13.510)	0.106
Child-Pugh class	A				
	B	0.000 (0.000– )	0.999		
AFP	<50				
	≥50	1.627 (0.435–6.085)	0.469		
Tumor size	<80 mm				
	≥80 mm	1.097 (0.159–7.567)	0.925		
Multiplicity	Single				
	Multiple	6.165 (1.229–30.933)	0.027	5.976 (1.359–26.274)	0.018
Vascular invasion	Absent				
	Present	0.322 (0.040–2.598)	0.287		
Microspheres	Resin				
	Glass	0.477 (0.088–2.585)	0.391		
Dose	<400	7.240 (1.204–43.521)	0.031	4.713 (1.281–17.336)	0.020
	≥400				

CR, complete response; AFP, alpha-fetoprotein; CI, confidence interval.

## Discussion

In this study, the objective response rate (CR+PR) was 59.2%, and the CR rate was 24.9%. Although the compositions of the patient groups, including disease stage, were highly heterogeneous, the outcomes were comparable with those of recently published studies, presenting 55–70.9% objective response rates (20–22). Patients with lower tumor marker levels, smaller tumors, single tumors, no vascular invasion, and those who received higher radiation doses showed better outcomes, which also corresponded with the results from previous studies (10–12, 23).

Recurrence after the achievement of CR after radioembolization for HCC is not uncommon (38.4%), and over 40% of recurrences occurred at and around the treated lesion. Two-thirds of the recurrences occurred within 8 months after the determination of CR, though recurrence can occur as late as 57 months after CR. These results imply that close and long-term follow-up is needed for HCC patients showing good responses to radioembolization.

Similar to the factors that influence the outcomes of radioembolization, the presence of multiple tumors and low target radiation doses were independently associated with an increased risk of recurrence after CR. Given their higher likelihood of recurrence, patients with multiple tumors or those who receive lower target radiation doses require closer monitoring. Based on the physician’s judgment, more proactive supplementary treatments should be considered for these patients.

Surgical resection and transplantation are curative and the best treatment options for HCC treatment. It has been reported that good therapeutic outcomes can be achieved when these surgical treatments follow bridging or down-staging with radioembolization (13–16). In this series, eight patients underwent curative resection or transplantation while in a CR status without any recurrence. Moreover, none of these patients experienced a recurrence during the median follow-up period of over 15 months. Thus, for patients with risk factors of recurrence after achieving CR, surgical treatment may be considered as a proactive supplementary treatment option. However, given the limited number of patients included in our study, definitive conclusions cannot be drawn. Further research is necessary to identify the appropriate patient group, determine the optimal time for surgery, and assess treatment outcomes.

Numerous studies have reported dose-response relationships. Several studies, including the LEGACY and DOSISPHERE studies, have confirmed that a higher radiation dose is an important factor in achieving better clinical and histological responses (24–28). In our study, higher mean radiation doses showed significant results in inducing and maintaining CR. If clinically feasible, selective infusion techniques, such as radiation segmentectomy, are essential for delivering higher radiation doses than conventional lobar infusion techniques. These approaches maximally spare the non-involved hepatic parenchyma and deliver higher radiation doses of at least 400 Gy in the case of glass microspheres and at least 200 Gy in the case of resin microspheres, with minimal adverse effects (29–31). When planning radioembolization, it is necessary to maximize the dose within a safe range.

This study has several limitations. The results may be biased and difficult to generalize due to the retrospective design of the study, and the results are derived from single-center data. To secure the required number of patients for analysis, the patient group in which CR was maintained for over 1 year was selected for the logistic regression analysis to determine the factors influencing the risk of recurrence after CR, considering that over two-thirds of recurrence cases occurred within 1 year. Nevertheless, the numbers of patients corresponding to each group (CR and non-CR) are not large, and as recurrence is not uncommon even more than 1 year following CR, the value of the results of this study may be limited.

In conclusion, the recurrence of HCC following CR after radioembolization is not uncommon and frequently occurs within 1 year after CR. Multiple tumors and lower target radiation doses may be risk factors for recurrence.

## Data availability statement

The raw data supporting the conclusions of this article will be made available by the authors, without undue reservation.

## Ethics statement

The studies involving humans were approved by Human Research Protection Center, Severance Hospital, Yonsei University Health System. The studies were conducted in accordance with the local legislation and institutional requirements. The participants provided their written informed consent to participate in this study.

## Author contributions

SM: Data curation, Formal analysis, Investigation, Resources, Writing – original draft. GK: Conceptualization, Methodology, Project administration, Resources, Supervision, Validation, Writing – review & editing. JW: Conceptualization, Project administration, Resources, Supervision, Validation, Writing – review & editing. JK: Formal analysis, Investigation, Methodology, Resources, Writing – review & editing. JP: Data curation, Investigation, Resources, Writing – review & editing. KH: Formal analysis, Investigation, Methodology, Writing – review & editing. MK: Conceptualization, Supervision, Writing – review & editing. HK: Data curation, Investigation, Writing – review & editing. DK: Data curation, Investigation, Writing – review & editing. JC: Data curation, Investigation, Writing – review & editing.
